# Manual Acupuncture Suppresses the Expression of Proinflammatory Proteins Associated with the NLRP3 Inflammasome in the Hippocampus of SAMP8 Mice

**DOI:** 10.1155/2017/3435891

**Published:** 2017-08-20

**Authors:** Ning Ding, Jing Jiang, Menghan Lu, Jiatong Hu, Yiyuan Xu, Xiaoxiao Liu, Zhigang Li

**Affiliations:** Beijing University of Traditional Chinese Medicine, Beijing 100029, China

## Abstract

**Objective:**

To investigate the effect of manual acupuncture (MA) on NLRP3 inflammasome-related proteins.

**Methods:**

SAMP8 mice were randomly divided into Alzheimer's disease (AD) group, the MA group, and the medicine (M) group. Mice in the M group were treated with donepezil hydrochloride at 0.65 *μ*g/g. In the MA group, MA was applied on Baihui (GV20) and Yintang (GV29) for 20 min and then pricked at Shuigou (GV26). The Morris water maze was applied to assess spatial learning and memory. Immunohistochemical staining and western blot analysis were used to observe the expression of NLRP3 inflammasome-related proteins.

**Results:**

Compared with the normal (N) control group, spatial learning and the memory capabilities of the AD group significantly decreased (*p* < 0.01). The number of NLRP3, ASC, Caspase-1, and IL-1*β* positively stained cells in the AD group was higher than the N group, and the relative expression levels of the above proteins were significantly higher than those in the N group (*p* < 0.01). These changes were reversed by both MA and donepezil (*p* < 0.01).

**Conclusion:**

MA can improve the learning and memory capabilities of SAMP8 mice. The negative regulation of the NLRP3/Caspase-1 pathway in the hippocampus may be a possible mechanism of MA in the treatment of AD.

## 1. Introduction

Alzheimer's disease (AD) is a type of neurodegenerative disease that is primarily characterized by progressive amnesia and has a high incidence among people over the age of 65. Collected data show that, in 2015, 46 million people lived with dementia worldwide; this number is estimated to increase to 131.5 million by 2050 [1]. This incurs a heavy burden for the social and economic development of a country and has already become the most urgent public health problem of the 21st century [1]. Traditional Chinese medicine has a long history and has been extensively used to treat dementia. In particular, many clinical and experimental studies have established that acupuncture plays a vital role in preventing and controlling AD due to its advantages of safety, convenience, and low side effects. A meta-analysis not only supports the high safety of the technique but also indicates that acupuncture is more effective than drugs at improving AD patients' ability to carry out their daily lives and may even enhance the effects of drug treatments [2]. fMRI studies have shown that acupuncture can enhance hippocampal connectivity [3], modulate default mode network activity [4], and activate certain cognitive-related regions in AD patients [5]. The positive effect of acupuncture on blood perfusion and glycol metabolism in certain brain areas in a rat model of AD has also been certified by PET studies [6, 7]. Furthermore, enhancing antioxidation in the hippocampus [8], reversing the upregulation of astrocytic NDRG2 [9], improving mitochondrial biogenesis and energy metabolism [10], and modulating the Wnt signal transduction pathway [11] have been confirmed as mechanisms by which acupuncture exerts its therapeutic effects during the treatment of AD.

Evidence from epidemiologic and fundamental studies on AD has established that immune system-mediated actions contribute to the process of neuroinflammation and drive AD pathogenesis [12, 13]. Microglia, which are the principal immune effector cells in the nervous system, play a key role in activating inflammatory responses in the AD brain [14]. As a potent stimulus for the inflammatory responses of microglia, amyloid-beta (A*β*) promotes an inflammatory response that is mediated by microglia and other immune cells, thus activating signaling pathways that could lead to neurodegeneration [15, 16]. In this process, the NLRP3 inflammasome, which is a molecular target for neuroprotection and therapeutic intervention in AD [17] and is the most extensively studied member of the NLR family [18], plays a pivotal role in the immune response to A*β*. The NLRP3 inflammasome is a large cytoplasmic multiprotein complex that promotes the recruitment of procaspase-1 via the ASC adaptor protein [19]. The subsequent activation of Caspase-1 by NLRP3 can promote the secretion of IL-1*β*, which is a potent proinflammatory factor [20]. Our previous researches show that acupuncture has a benign regulatory effect on A*β* in the brain that includes decreasing the expression of A*β* in the hippocampus and its content in the frontal lobe [21, 22]. However, until now, the effects of manual acupuncture (MA) intervention on the NLRP3 inflammasome and its associated proteins in AD cases remain largely elusive. Therefore, the goal of this study is to elucidate the MA effect on NLRP3 inflammasome-related proteins and further clarify the mechanism of MA in improving neuroinflammation in AD cases. This is the first time, to the best of our knowledge, that a study has focused on the NLRP3 inflammasome response after MA treatment in an attempt to elucidate the underlying mechanisms of action of MA in 8-month-old SAMP8 mice by immunohistochemical staining and western blot analysis.

## 2. Materials and Methods

### 2.1. Experimental Animals

Senescence-accelerated mouse prone 8 (SAMP8) and the normal cognate senescence-accelerated mouse-R1 (SAMR1) mice strains were purchased from the Experimental Animal Center of First Teaching Hospital of the Tianjin University of Traditional Chinese Medicine (Animal Lot: SCXK(Jin)2013-0001). Both types of mice weighed 30.0 ± 2.0 g and were 8 months old. The animals were housed in a fenced facility in the Experimental Animal Center of the First Teaching Hospital of Beijing University of Traditional Chinese Medicine at a controlled temperature (24 ± 2°C) and under a 12-h dark/light cycle, with sterile drinking water and a standard pellet diet available ad libitum. All mice were acclimatized to the environment for 7 days prior to experimentation, and all experimental procedures complied with the guidelines of the “Principles of Laboratory Animal Care” formulated by the National Institute of Health and the legislation of the People's Republic of China for the use and care of laboratory animals.

### 2.2. Animal Grouping and Intervention

Twenty-four 8-month-old SAMP8 male mice were divided into three groups (*n* = 8 per group): Alzheimer's disease control (AD) group, the manual acupuncture (MA) group, and the medicine group (M) group. Eight 8-month-old SAMR1 male mice were used as the normal control (N) group.

In the MA group, the mice were immobilized in mouse bags. MA on Baihui (GV20) and Yintang (GV29) was applied for 20 min, with transverse puncturing at a depth of 2-3 mm; then, the mice were pricked at Shuigou (GV26) with disposable sterile acupuncture needles (0.25 mm × 13 mm) (Beijing Zhongyan Taihe Medicine Company, Ltd). During the MA on Baihui (GV20) and Yintang (GV29), twirling manipulation was applied every 5 min and lasted 15 s each time. Each needle was rotated bidirectionally within 90° at a speed of 180°/s. The selection of the acupoints was based on findings from our previous studies [23, 24]. For the M group, donepezil hydrochloride tablets (Eisai China Inc., H20050978) were crushed and dissolved in distilled water and were delivered to mice by oral gavage at a dose of 0.65 *μ*g/g [25]. The above treatments were administered once a day for 15 consecutive days, but no treatment was carried out in the N or AD groups. The mice in the AD, N, and M groups received the same 20-min restriction as the MA group.

### 2.3. The Morris Water Maze Test

Morris' water maze consisted of a circular tank (diameter, 90 cm; height, 50 cm) filled with water to a depth of 30 cm, was maintained at 24 ± 1°C, and was rendered opaque with blue-black ink. A removable circular platform (diameter, 9.5 cm; height, 28 cm) with the top surface 1 cm below the water was located inside the pool. The pool area was conceptually divided into four quadrants (NE, NW, SW, and SE) of equal size. Visual cues of different shapes were placed on the tank wall of each quadrant in plain sight of the mice. The experiment room was designed to maintain sound insulation, with an indirect light source and a low-light environment, and the remaining objects in this room were kept in their original locations. The experimental conditions were unchanged for the duration of the test. The data were automatically collected by a video camera (TOTA-450d, Japan) that was fixed to the ceiling and connected to a video recorder with an automated tracking system (China Daheng Group, Beijing, China). To test the behavior of spatial learning and memory, each mouse underwent a 5-day hidden platform trial and then a 1-day probe trial.

#### 2.3.1. Hidden Platform Trial

Three locations in quadrants I, II, and IV, which were equidistant to the center of the tank, were selected as entry points. Each mouse was released from one of three entry points and had 60 s to search for the hidden platform. At the end of each trial, the mouse was placed on the platform or allowed to stay there for 10 s. Six trials per day for 5 consecutive days were performed, with the visual cues kept constant. The time that a mouse took to find the platform was recorded and represented escape latency.

#### 2.3.2. Probe Trial

The day after the completion of the hidden platform test, the platform was removed. Each mouse was placed in the pool once for 60 s, starting from the same initial location used in the hidden platform test. The platform crossover number and swimming distance in the platform quadrant were recorded, and the percentage of the swimming distance in the platform quadrant was derived.

### 2.4. Immunohistochemical Staining

The brains of 2 mice from each group were fixed in paraformaldehyde after cardiac perfusion and were then trimmed, dehydrated with ethanol, made transparent with xylene, embedded in paraffin, and sectioned on a coronal plane with a 6 *μ*m slicer. Subsequently, the sections were dewaxed and hydrated and incubated first for 5 min with 0.01 mol/L of citrate buffer for antigen thermal remediation and then for 10 min with 3% methanol hydrogen peroxide at room temperature. Next, the sections were blocked in 2% BSA for 10 min and incubated with primary antibody diluent (Beijing, Biorbyt, NLRP3, 1 : 500; USA, NOVUS, ASC, 1 : 900; USA, ABCAM, Caspase-1, 1 : 600; USA, ABCAM, IL-1*β*, 1 : 50) for 12 h at 4°C. Then, the sections were rinsed with phosphate-buffered saline (PBS) and incubated with secondary antibody diluent (Shanghai, Jiehao, Haopoly-HRP, 1 : 1000) for 30 min at 37°C. The sections were rinsed with PBS and placed into diaminobenzidine (DAB) solution for 5 minutes after being rinsed another time with PBS. After being redyed with hematoxylin, the brain slices were dehydrated and observed under a light microscope, BX53 (Olympus Corporation, Japan).

### 2.5. Western Blot Analysis

The remaining mice in each group were sacrificed under anesthesia to harvest their hippocampi. After liquid nitrogen extraction and protein extraction, SDS-PAGE electrophoresis was performed with a 10% separating gel and a 5% stacking gel and transferred to a 0.45-*μ*m PVDF membrane. Membrane blocking was performed using 5% nonfat milk in Tris-buffered saline supplemented with 0.1% Tween 20 (TBST). The primary antibody (Beijing, Biorbyt, NLRP3, 1 : 300; USA, NOVUS, ASC, 1 : 500; USA, ABCAM, Caspase-1, 1 : 1000; USA, ABCAM, IL-1*β*, 1 : 2500) was added, followed by incubation for one night at 4°C. The secondary antibody (Shanghai, Jiehao, Haopoly-HRP, 1 : 5000) was added before shaking and incubating at room temperature for 1 h. HRP-ECL luminous liquid was added, and X-ray film exposure was completed in a dark room following developing and fixing. After calibrating the control markers, the scanning and analysis were performed by Quantity One, and the relative expressions of NLRP3, ASC, Caspase-1, and IL-1*β* were compared in each group.

### 2.6. Statistical Analysis

The statistical analysis was performed using the SPSS software, version 17.0 (SPSS, Inc., Chicago, IL, USA), and the data were expressed as the mean ± standard deviation. A one-way ANOVA was used after the normal distribution and homogeneity of variance were confirmed. For the non-normally distributed data or for data with heterogeneous variance, a nonparametric test was used. The LSD method was applied for pairwise comparisons of the western blot results. Statistical significance was set to *p* < 0.05 and high statistical significance was set to *p* < 0.01.

## 3. Results

### 3.1. Effect of MA on Spatial Learning and Memory

**Figure 1:**
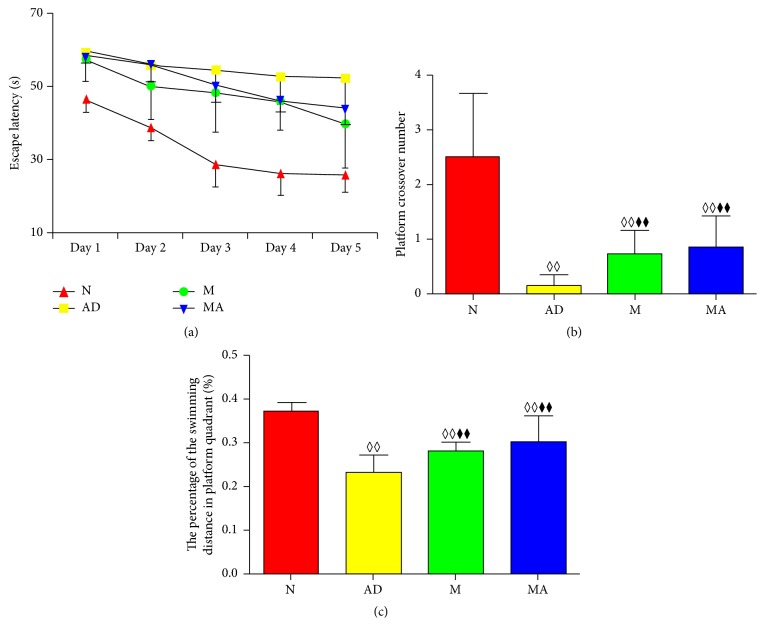
Comparison of the learning and memory behavioral testing in each group. (a) The trend in escape latency in all groups. (b) Comparison of the platform crossover numbers of all groups. (c) Comparison of the percentage of the swimming distance in the platform quadrant of all groups. ^◊◊^*p* < 0.01, ^◊^*p* < 0.05 compared with the N group. ^⧫⧫^*p* < 0.01, ^⧫^*p* < 0.05 compared with the AD group. ^△^*p* < 0.05 compared with the M group.

The results of the Morris water maze test are presented in Table 1 and Figure 1. The escape latency, platform crossover number, and percentage of the swimming distance in the platform quadrant were significantly higher in the AD, M, and MA groups than in the N group (*p* < 0.01). Escape latency in the N, M, and MA groups decreased gradually from day 1 to day 5, but the AD group maintained a high value. Compared with the AD group, escape latency in the M and MA groups on day 5 decreased significantly (*p* < 0.05). The platform crossover number and percentage of the swimming distance in the platform quadrant of the M and MA groups increased significantly compared with the AD group (*p* < 0.01).

### 3.2. Effect of MA on NLRP3 Inflammasome-Related Proteins

**Figure 2:**
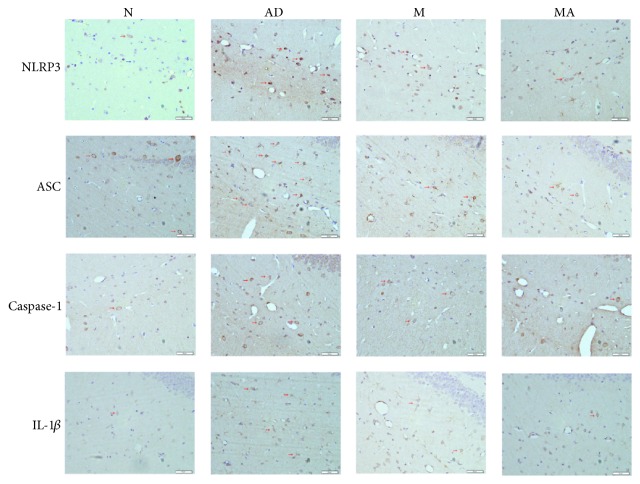
Light microscopy imaging (400x) of mice brain slices, immunohistochemically stained with antibodies specific for the detection of NLRP3, ASC, Caspase-1, and IL-1*β*. The positively stained cells appear brown (red arrow), and the negatively stained cells are blue (blue arrow).

Immunohistochemistry images of NLRP3-, ASC-, Caspase-1- and IL-1*β*-stained hippocampal brain slices are presented in Figure 2. The results showed that the above proteins were mainly distributed in the membrane and the cytoplasm of the positively stained cells. In the N, M, and MA groups, there were fewer positively stained cells, and they were weakly stained. The NLRP3, ASC, Caspase-1, and IL-1*β* positively stained cells clearly displayed processes and showed obvious increases in both their numbers and level of staining in the AD group.

### 3.3. Effect of MA on the Relative Expression of NLRP3 Inflammasome-Related Proteins

**Figure 3:**
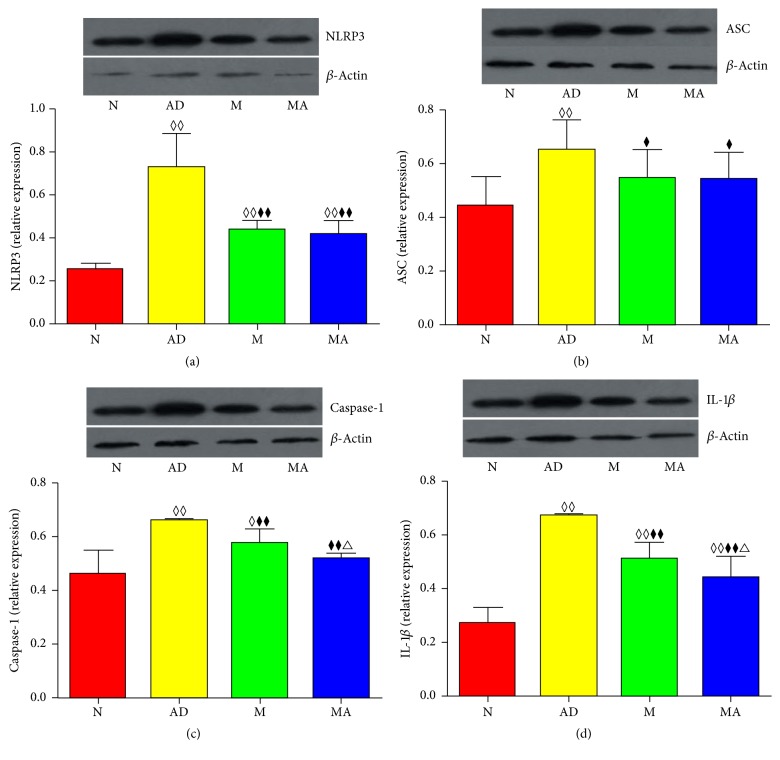
Comparison of the relative expressions and immunoblot levels of (a) NLRP3, (b) ASC, (c) Caspase-1, and (d) IL-1*β* in each group after 15 consecutive days of MA treatment. ^◊◊^*p* < 0.01, ^◊^*p* < 0.05 compared with the N group. ^⧫⧫^*p* < 0.01, ^⧫^*p* < 0.05 compared with the AD group. ^△^*p* < 0.05 compared with the M group.

The western blotting results of NLRP3, ASC, Caspase-1, and IL-1*β* in the hippocampus are shown in Figure 3. Compared with the N group, the relative expressions of NLRP3, ASC, Caspase-1, and IL-1*β* significantly increased in the AD (*p* < 0.01), MA (*p* < 0.01), and M groups (*p* < 0.01 or 0.05), with the exception of ASC and Caspase-1, which did not show significant differences in the MA group (*p* > 0.05). The relative expressions of the above proteins in the MA and M groups were lower than those in the AD group (*p* < 0.01 or 0.05). The comparative analysis between the MA and M groups showed that the relative expression levels of Caspase-1 and IL-1*β* were drastically lower in the MA group than in the M group (*p* < 0.01).

## 4. Discussion

### 4.1. MA Significantly Improves the Spatial Learning and Memory of SAMP8 Mice

As one of the most common tasks used to assess spatial learning and memory ability in rodents [26], the Morris water maze was used in this study. The hidden platform trial and probe trial were used to assess the capabilities in spatial learning and memory, respectively. The results showed that 8-month-old SAMP8 mice have characteristic learning and memory deficits, which indicates that SAMP8 mice are ideal animal models for studying AD. Furthermore, both donepezil and MA can improve the spatial learning and memory ability of SAMP8 mice after 15 consecutive days, and their effects are identical.

### 4.2. NLRP3 Inflammasome Plays a Pivotal Role in the Inflammatory Response Mediated by Microglia in AD

The NLRP3 inflammasome is a large cytoplasmic multiprotein complex (>700 kDa) that is composed of the NLRP3 (nucleotide-binding domain and leucine-rich repeat protein 3) and the ASC (apoptosis-associated speck-like protein containing a CARD domain) proteins [19]. NLRP3 is responsible for the formation of inflammasomes and the activation of procaspase-1 through its pyrin domain, and the ASC protein promotes the recruitment of procaspase-1 [27]. Therefore, the NLRP3 inflammasome provides a molecular platform for the activation of Caspase-1, which can then regulate the maturation and secretion of IL-1*β* and IL-18, thereby significantly affecting innate and acquired immunity [28]. Activated Caspase-1 can mediate the proteolytic cleavage of pro-IL-1*β* into IL-1*β* [29]. Studies have shown that the NLRP3 inflammasome is required for the A*β*-induced activation of Caspase-1, the release of mature IL-1*β*, and the secretion of proinflammatory and potentially neurotoxic cytokines and chemokines [30]. NLRP3^−/−^ or Caspase-1^−/−^ mice showed reduced brain Caspase-1 and IL-1*β* activation, protected spatial memory, and enhanced A*β* clearance [31], indicating that the activation of the NALP3 inflammasome by A*β* may be a critical component of the inflammatory response in AD. In particular, A*β* promotes the formation of the NLRP3 inflammasome in microglia, thus leading to the activation of procaspase-1 and the secretion of IL-1*β*, ultimately resulting in an inflammatory response in the brain and inducing neuronal necrosis and apoptosis [32–35]. Collectively, the above studies demonstrate the important role of the NLRP3/Caspase-1 pathway in the pathogenesis of AD.

### 4.3. MA Suppresses the Elevated Expression of NLRP3 and IL-1*β* in AD

Our results showed that NLRP3, ASC, Caspase-1, and IL-1*β* positively stained cells in the AD group became more abundant and that their expression increased significantly (*p* < 0.01) compared with the N group. These findings indicate that the inflammatory response induced by microglia in the hippocampus of 8-month-old SAMP8 mice undergoes similar pathological changes to the brains of AD patients. After 15 consecutive days of MA treatment, improvements in all measured parameters were observed, demonstrating that MA can exert its anti-inflammatory effect by decreasing the expression of NLRP3 inflammasome-related proteins and by negatively regulating the NLRP3/Caspase-1 pathway. Suppressing the neuroinflammatory responses induced by the NLRP3 inflammasome and downregulating the maturation and secretion of IL-1*β* in the hippocampus may be some of the mechanisms by which MA acts against AD. Combined with our prior results on A*β* [21, 22] and its relationship with NLRP3 inflammasome we mentioned above, benign regulative effect on A*β* and its downstream NLRP3/Caspase-1 pathway can be seen as the important mechanism of MA against AD.

### 4.4. Effect and Mechanism of MA Are Distinct from the Donepezil Treatment of AD

Our study revealed that donepezil has the same anti-inflammatory effect on the NLRP3/Caspase-1 cascade as MA, which is consistent with previous reports [36, 37]. However, there were differences in the magnitude of this effect. In particular, our results indicated that MA was more effective in downregulating Caspase-1 and IL-1*β* than donepezil; however, there were no differences in the expressions of NLRP3 and ASC. Notably, research has not identified a clear association between the level of ASC and Caspase-1, and the latter can be regulated by alternative factors, such as NF*κ*B [38] and Nedd8 [39]. Moreover, increasing evidence suggests that inflammasomes and Caspase-1 are not the only mechanisms for the processing of the IL-1 family [40]. Consequently, the superior effect of MA on Caspase-1 and IL-1*β* implied that MA exerts its AD treatment effects by targeting multiple pathways, which conforms to the general basis of acupuncture. Overall, our study demonstrates that MA has a superior effect compared to donepezil in relieving the anti-inflammatory response mediated by the inflammasome. Whether the effect of MA on the treatment of AD is mediated via a multiregulatory network of anti-inflammatory responses warrants further investigation in the future.

### 4.5. Future Research Prospects

Although both MA and donepezil suppressed the increased expression of inflammasome-related inflammatory proteins, this suppression was not complete, which indicated that additional pathways may be involved. This emphasizes the complexity of AD treatment and the importance of early intervention. In addition, a failure to fully suppress the inflammatory response may be attributed to limitations of the MA treatment. Further studies in the future, with larger sample sizes and adjustments of the treatment duration, would improve our understanding of the signaling network regulated by MA treatment. In addition, an accurate assessment of the protein levels by ELISA and genetic studies are critical for revealing the implicated pathways. Finally, identifying the mechanisms by which MA regulates the anti-inflammation network would optimize the therapeutic regimen for AD patients.

## 5. Conclusion

This study, for the first time and to the best of our knowledge, confirms that MA can decrease the expression of NLRP3 inflammasome-related proteins, thus contributing greatly to revealing the underlying mechanism of MA in treating AD. Additionally, our study further supports the effectiveness of MA in the treatment of AD by showing that the anti-inflammatory effect of MA is superior to donepezil treatment. In conclusion, MA has the scientific basis to be considered a promising therapeutic approach for treating AD. Considering its safety, low side effects, and convenience in use, it deserves to be more broadly available in clinical practice.

## Figures and Tables

**Table 1:** Comparison of escape latency in each group in the hidden platform trial (*x* ± *s*, s,  *n* = 8).

Group	Day 1	Day 2	Day 3	Day 4	Day 5
N group	46.63 ± 3.56	38.70 ± 3.46	28.58 ± 6.13	26.09 ± 5.89	25.69 ± 4.83
AD group	59.58 ± 1.18^◊◊^	56.07 ± 4.27^◊◊^	54.58 ± 6.13^◊◊^	52.94 ± 7.73^◊◊^	52.54 ± 8.15^◊◊^
M group	57.58 ± 6.05^◊◊^	50.29 ± 9.29^◊^	48.41 ± 10.92^◊◊^	46.06 ± 7.98^◊◊^	39.82 ± 12.15^◊⧫^
MA group	58.28 ± 1.77^◊◊^	56.21 ± 4.81^◊◊^	50.36 ± 4.71^◊◊^	46.52 ± 3.29^◊◊^	43.76 ± 4.12^◊◊⧫^

*Notes*. ^◊◊^*p* < 0.01, ^◊^*p* < 0.05 compared with the N group. ^⧫⧫^*p* < 0.01, ^⧫^*p* < 0.05 compared with the AD group. ^△^*p* < 0.05 compared with the M group.
